# Patient-focused pathogen genetic counselling—has the time come?

**DOI:** 10.1186/s13073-021-00993-w

**Published:** 2021-11-08

**Authors:** Angeline S Ferdinand, Jane S Hocking, Justin T. Denholm, Benjamin P. Howden, Deborah A. Williamson

**Affiliations:** 1 Microbiological Diagnostic Unit Public Health Laboratory, Department of Microbiology and Immunology, The University of Melbourne at The Peter Doherty Institute for Infection and Immunity, 792 Elizabeth Street, Melbourne, 3100 Australia; 2 Melbourne School of Population and Global Health, University of Melbourne, Melbourne, Australia; 3 Victorian Tuberculosis Program, Melbourne Health, Melbourne, Australia; 4 Department of Infectious Diseases, The University of Melbourne at The Peter Doherty Institute for Infection and Immunity, 792 Elizabeth Street, Melbourne, 3100 Australia; 5 Present Address: Department of Microbiology and Immunology, The University of Melbourne at The Peter Doherty Institute for Infection and Immunity, 792 Elizabeth Street, Melbourne, Australia; 6 Victorian Infectious Diseases Reference Laboratory, Royal Melbourne Hospital at The Peter Doherty Institute for Infection and Immunity, Melbourne, Australia

## Abstract

Ensuring accordance with principles of healthcare ethics requires improved communication of pathogen genomic data. This could include educating healthcare professionals in communicating pathogen genomic information to individuals, developing ethical frameworks for reporting pathogen genomic results to individuals, responsible media reporting guidelines, and counselling for individuals (‘pathogen genetic counselling’).

## Background

Pathogen genome sequencing is a powerful tool that can be used to track the emergence and spread of infectious diseases across the globe, as illustrated by the COVID-19 pandemic. Sequencing of SARS-CoV-2 genomes has been integral to the public health response since the start of the pandemic, from development of the first diagnostic tests, through to management of outbreaks, design of vaccines and the detection of novel, more transmissible viral variants [1]. The use of genome sequencing has also entered mainstream public and political discourse, informing government policy around public health measures such as lockdowns and social distancing and, in the case of SARS-CoV-2 variants, guiding restrictions on international travel [2].

In some settings, pathogen genomics has informed public health responses to infectious diseases for several years. For example, in the USA, pathogen genome sequencing now forms the cornerstone of foodborne disease surveillance, providing critical information on the sources and transmission of foodborne pathogens [3]. Similarly, in the UK and Australia, prospective whole genome sequencing (WGS) of *Mycobacterium tuberculosis* isolates has been underway for several years, uncovering previously occult transmission networks of tuberculosis (TB) and informing epidemiological analysis [4, 5]. Indeed, the scope of infections in clinical and public health settings where WGS is now in use is so broad that it spans the gamut of human interactions, from breathing (e.g. COVID-19; TB), to eating (e.g. salmonellosis), to sexual activities (e.g. gonorrhoea). Compared with previous methods used to assess relatedness of pathogens (e.g. multilocus sequence typing; pulsed-field gel electrophoresis), the insights afforded by WGS provide a much higher degree of confidence in inferring relationships between pathogens, delineating clusters of disease, identifying sources of outbreaks and transmission pathways. When utilised in public health practice to identify and interrupt disease outbreaks, pathogen genomic data generated from individuals are generally considered collectively, as a potentially related group of sequences, rather than on an individual basis. Importantly however, a fundamental requirement for the generation of such collective data is an individual diagnosis of infection (or colonisation)—that is, underlying each pathogen sequence is an individual patient, with a specific social and behavioural context for their infection.

## How are genomic results communicated to individuals?

For most infectious diseases, a diagnostic test result is provided back to patients, usually by a healthcare professional. Depending on the pathogen, there may be specific guidelines for communicating this diagnosis to individuals, with established pathways into treatment and ongoing healthcare. For example, in the USA and other high-income settings, a diagnosis of HIV will generally trigger entry into a continuum of care, with referral to counselling and support services [6]. However, in the case of pathogen genomic data, to date, limited to no information on the genomic relational context is provided back to individuals diagnosed with the infection and indeed may not even be provided back to their healthcare practitioner. Put simply, communication to individuals around infectious diseases has generally focused on ‘What do I have?’ rather than ‘How or where did I get it?’

In the case of genetic factors relating to non-communicable diseases, there are established pathways relating to the sharing of knowledge back to individuals, within the framework of genetic counselling services. Such counselling enables a process of bidirectional information sharing, provides ongoing support for individuals, and places their illness within a broader societal and behavioural context. However, compared to the decades of work developing ethical frameworks for reporting human genetic disorders, reporting of pathogen genomic data (e.g. detection of in silico virulence mechanisms potentially impacting disease manifestations; links to other pathogen genomes suggesting a possible source of infection / transmission) to individuals is in its infancy. To date, most work assessing the ethical implications of pathogen sequencing has been conducted in the field of HIV phylogenetic research, where the potential benefits and harms of utilising genomic data have been well-described [7]. A major impetus for developing an ethical framework for HIV molecular epidemiological work has been the potential for criminalisation of phylogenetic results and other adverse consequences such as stigmatisation and marginalisation of specific population groups [7].

## Lessons from COVID-19

The COVID-19 pandemic has placed the issue of ‘precision pathogen genomics’ reporting into sharp relief. In many countries, the groups most affected by COVID-19 are frontline workers (e.g., healthcare workers, border staff or individuals working in closed settings) and vulnerable populations (e.g. aged care). For many of these individuals and their families, it is inevitable that questions will arise regarding potential links to genomic clusters (e.g., occupational exposures; healthcare-associated infections), and it is possible requests for such genomic information may extend into the coronial and judicial systems. These issues may be particularly acute in low-prevalence countries such as Australia and New Zealand, where there is a high degree of SARS-CoV-2 sequencing coverage [8]. In these settings, new infections are often announced in the media, with accompanying public information about genomic sequencing results (e.g. whether the individual has a variant of concern, or which cluster the patient is linked to). In this context, it is possible that individuals and their contacts first hear about genomic information relating to their infection through the media, rather than from a trained healthcare professional. This can threaten the privacy of the individual, impact negatively on their social and professional networks, and may actually deter individuals in the community from testing. This combination of heightened concern and uncertainty regarding more appropriate approaches to disclosure of genomic information may also deter clinicians from direct communication of results and their significance to the individual.

## What issues should we consider when reporting pathogen genomic results?

To ensure the implementation of pathogen genomics is in accordance with the overarching principles of healthcare ethics, and balances public health benefit with individual autonomy and privacy, we believe an improved focus on communication of pathogen genomic data by healthcare professionals is required to individuals, policymakers, the media, and the wider community. As a first step, this could involve education of healthcare professionals regarding the strengths and limitations of genomic data (i.e., what the data can and cannot show) and when and how to communicate this information to individuals and their families. Second, major national and international agencies deploying pathogen genomics at the clinical and public health interface could collectively develop and implement an ethically grounded framework for reporting results from pathogen genomic data, addressing some of the major questions that individuals with an infection may have (e.g. ‘where did I get my infection?’; ‘how many other people have this infecting strain?’). Establishment of this framework could be facilitated by the formation of a multinational working group, with appropriate consumer and end-user representation. This group could also develop ‘best-practice’ guidelines for the responsible reporting of pathogen genomics by media outlets to protect the privacy of individuals and minimise any adverse consequences for infected individuals and their networks and communities. Finally, we suggest that, depending on the genomic and epidemiological context of their infection, ongoing counselling and psychosocial support may be required for individuals and their families (‘pathogen genetic counselling’). This is particularly relevant if genomic analysis reveals a potentially sensitive or stigmatising context for their infection (e.g., COVID-19 infection in an aged care setting; acquisition of a multi-resistant organism in hospital; the index case for introduction of a novel variant into a new setting). Where genomic and epidemiological data suggest transmission within a particular community, public health initiatives to provide appropriate communication, education, and support to the community may also be warranted.

## Conclusions

Pathogen genomics has made huge technological and analytical advances over the past decade, culminating in widespread use for tracking the COVID-19 pandemic. In order to maintain public trust in agencies undertaking such work, the time has come to make sure that reporting of genomic data back to individuals and to the wider community is done in a timely, sensitive, and responsible manner, preferably by professionals who understand the strengths and shortcomings of the data. A pragmatic approach is required to balance the need to utilise and share genomic data (including relevant epidemiological data) in clinical and public health practice and the need to protect individuals and communities from unintended consequences and harm.

## Data Availability

Not applicable.
